# A global reconnaissance of particulates and metals/metalloids in untreated drinking water sources

**DOI:** 10.1007/s10661-021-09086-y

**Published:** 2021-04-28

**Authors:** Jonathan W. Peterson, Benjamin M. Fry, Daniel R. Wade, Ford J. Fishman, Jacob T. Stid, Jonas M. Peterson, Cleveland E. Tarp, Randall D. Wade, Sarah A. Brokus, Michael J. Pikaart, Brent P. Krueger, Aaron A. Best

**Affiliations:** 1 Department of Geological & Environmental Sciences, Hope College, Holland, MI USA; 2 Department of Chemistry, Hope College, Holland, MI USA; 3 Department of Biology, Hope College, Holland, MI USA

**Keywords:** Global water reconnaissance, Metal/metalloid contamination, Point-of-use water filters, Untreated-drinking water

## Abstract

Metal and metalloid contamination in drinking water sources is a global concern, particularly in developing countries. This study used hollow membrane water filters and metal-capturing polyurethane foams to sample 71 drinking water sources in 22 different countries. Field sampling was performed with sampling kits prepared in the lab at Hope College in Holland, MI, USA. Filters and foams were sent back to the lab after sampling, and subsequent analysis of flushates and rinsates allowed the estimation of suspended solids and metal and other analayte concentrations in source waters. Estimated particulate concentrations were 0–92 mg/L, and consisted of quartz, feldspar, and clay, with some samples containing metal oxides or sulfide phases. As and Cu were the only analytes which occurred above the World Health Organization (WHO) guidelines of 10 μg/L and 2000 μg/L, respectively, with As exceeding the guideline in 45% of the sources and Cu in 3%. Except for one value of ~ 285 μg/L, As concentrations were 45–200 μg/L (river), 65–179 μg/L (well), and 112–178 μg/L (tap). Other metals (Ce, Fe, Mg, Mn, Zn) with no WHO guideline were also detected, with Mn the most common. This study demonstrated that filters and foams can be used for reconnaissance characterization of untreated drinking water. However, estimated metal and other analyte concentrations could only be reported as minimum values due to potential incomplete retrieval of foam-bound analytes. A qualitative reporting methodology was used to report analytes as “present” if the concentration was below the WHO guideline, and “present-recommend retesting” if the concentration was quantifiable and above the WHO guideline.

## Introduction

Contamination of drinking water sources with dissolved metals is a growing global concern in developed countries (Chappells, 2015; Hanna-Attish et al., 2016; Harvey et al., 2016; Le Bot et al., 2016), and also particularly in the developing world where untreated sources are common (Bajwa et al., 2017; Dundar & Altundag, 2007; Karagas et al., 2015; Mohiuddin et al., 2011; Reza & Singh, 2010; Wyatt et al., 1998). Health effects from metal exposure are varied and comorbid, including osteomalacia from cadmium Cd (Yoshida et al., 1999); cognitive impairment from lead (Pb) (AAP, 2005); neurological, cardiovascular, and renal diseases from mercury (Hg) (Mamtani et al., 2011); immunotoxicity and pulmonary toxicity from nickel (Ni) (Das et al., 2008); and alimentary cancers from chromium (Cr) (Zhitkovich, 2011), to only mention a few. Arsenic (As) is possibly the most publically known metalloid contaminant on the global scale because of its widespread detection (Chowdhury et al., 2016) and its myriad human-health effects (Mamtani et al., 2011). In addition to the well-published crisis in Bangladesh (Chakraborti et al. 2015; Smith et al., 2000), it has been estimated that a minimum of about 140 million people in 50 countries have been drinking arsenic-contaminated water at levels above the World Health Organization (WHO) guideline value of 10 μg/L (WHO, 2018).

**Fig. 1:**
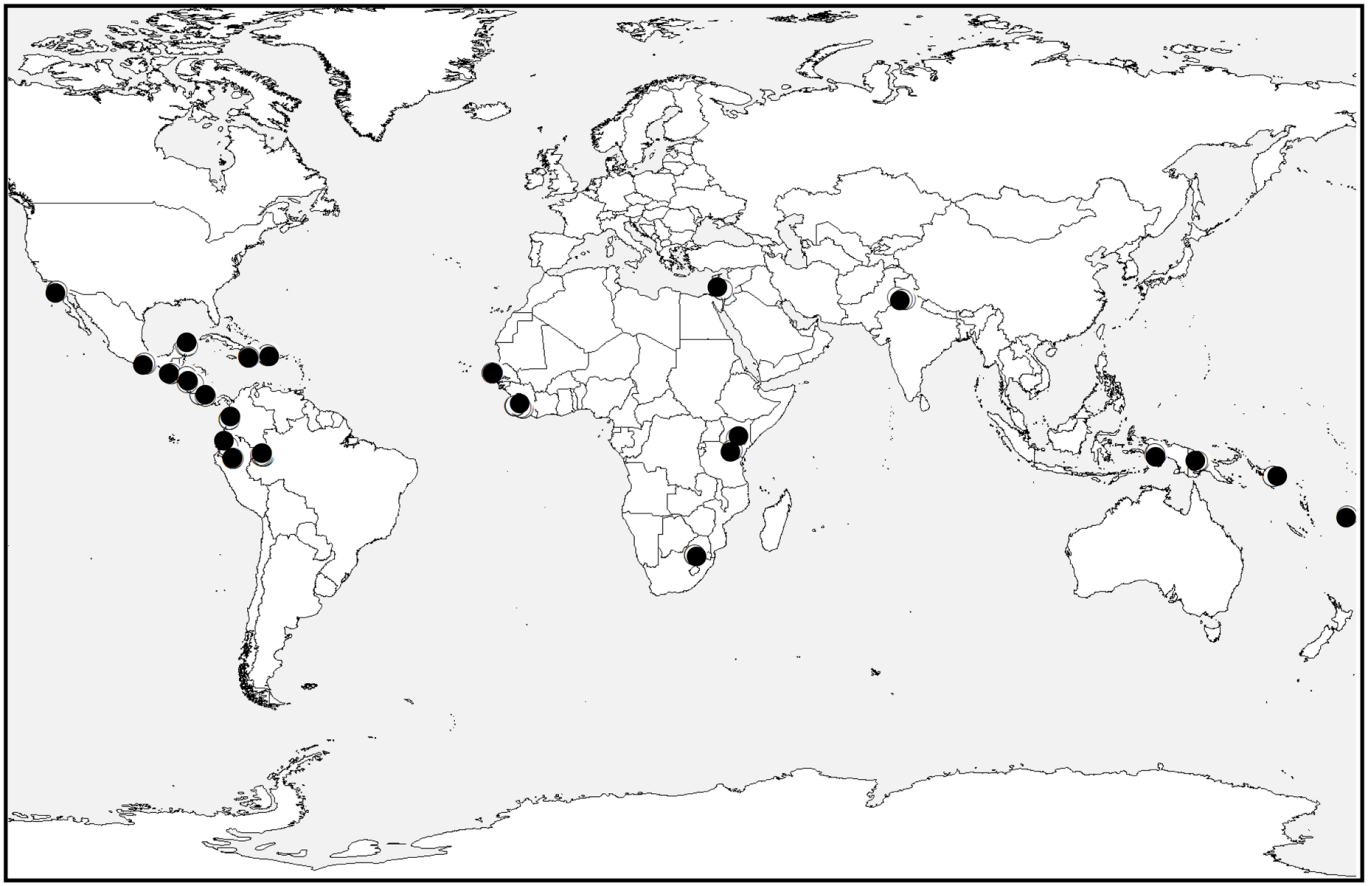
Map of countries where untreated drinking water was sampled in this study

In order to contribute to the understanding of the scope and characteristics of this world-wide metal contamination issue, this paper presents results from part of a global reconnaissance survey which investigated 71 untreated drinking water sources in 22 countries (Fig. 1) for the presence of dissolved metal contamination. Analytes included arsenic (As), barium (Ba), cadmium (Cd), cerium (Ce), chromium (Cr), copper (Cu), iron (Fe), magnesium (Mg), manganese (Mn), nickel (Ni), lead (Pb), antimony (Sb), selenium (Se), and zinc (Zn). Suspended load particulate matter can be a substrate and transport vehicle for metal, metalloid, and other contaminants (Rice et al., 2002; Dundar & Altundag, 2007; Alkhatib & Berna, 2008; Maniquiz-Redillas et al., 2014; Djukic et al., 2016; Nasrabadi et al., 2018; Yang et al., 2018); therefore, another part of this study was to collect concentration and composition data on suspended load from the same untreated drinking water sources tested for dissolved analytes.

Although many studies have been published on metal contamination of drinking water sources (Ćavar et al., 2005; Gowd & Govil, 2008; Mosaferi et al., 2008; Badr et al., 2011; Wongsasuluk et al., 2014; Chowdhury et al., 2016), this study was unique in several ways. First, it utilized an innovative method for testing water sources. This study eliminated the problem of shipping water internationally by capturing the water characteristics on a filter and foam, which were comparatively much easier and less expensive to transport. Second, samples were collected by the same protocol at all 71 sources, thereby minimizing variability due to field collection. Third, this was a reconnaissance tool with the purpose of identifying hotspot sites with high inorganic contaminant levels which could be targeted for subsequent, direct analysis of the water. This approach may avoid expensive analysis on waters which pose little to no long-term human health threat.

The work reported in this paper represents a two-part contribution, the project design and the results. The design was a new approach to providing water quality data to developing country stakeholders. The subsequent deliverable was reconnaissance data which can be easily communicated to technically-educated or non-technically trained decision-makers.

## Materials and methods

### Sampling

#### Field sampling-particulates/suspended load

**Fig. 2:**
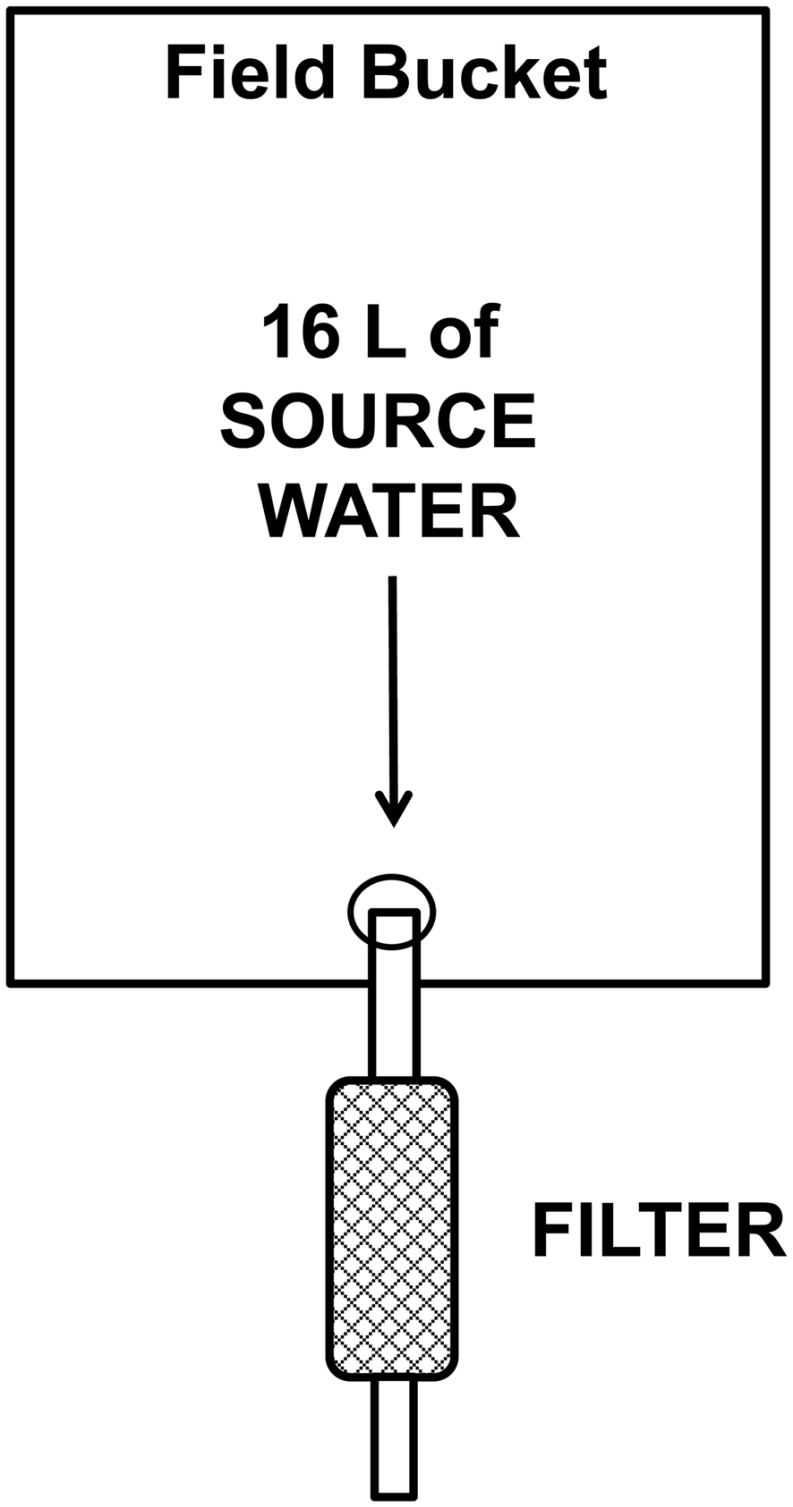
Schematic of bucket and filter sample collection method as used in the field

The foundation of this project was global field sampling of untreated drinking water sources performed by trained field staff and volunteers from several non-governmental organizations (NGOs). Sampling was done with 12-cm × 3.5-cm-diameter point-of-use 0.1-μm hollow fiber membrane filters (Sawyer Products, Inc.). Field kits containing filters, detailed instructions, and accessories to perform systematic sampling were assembled at Hope College in Holland, MI, USA, and sent to 71 locations in 22 countries. Water sources sampled included rivers, holding tanks, catchments, groundwater wells, wetlands, and household taps which conveyed untreated water. Sites were mostly rural or in small villages. New 18.9-L (5 gallon) plastic buckets were fitted with filters and tubing (Fig. 2). Buckets were rinsed with source water then filled with 16 L of source water which was allowed to gravity drain through the filter. After the bucket was drained, the filter was detached and four (50 mL) volumes of air were pushed through the sample with a syringe to flush out residual water. Four samples were collected in this manner at each site. Filters were then capped at both ends, placed in a zip-sealed plastic bag, and shipped back to Hope College.

#### Particulate retrieval

**Fig. 3:**
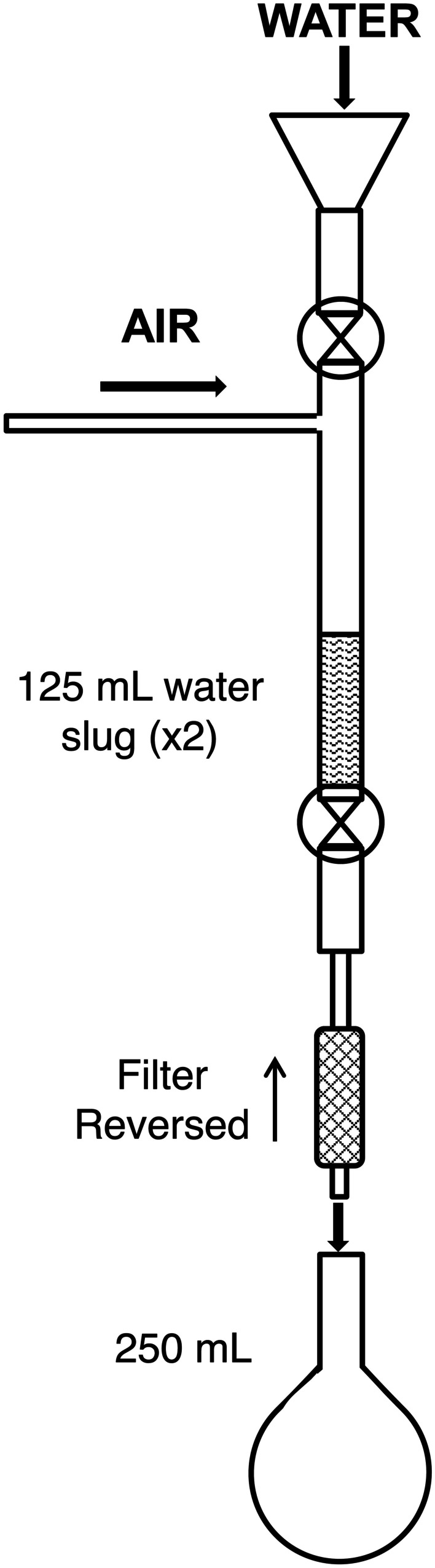
Schematic illustrating the laboratory back-flushing procedure to retrieve the suspended load particulates sampled in the field

An apparatus (Fig. 3) was designed, and a procedure was developed to backflush filters returned from the field in order to reclaim the source water particulates captured on the filter. The filter was attached to the apparatus in a reverse orientation. Two (125 mL) volumes of 18MΩ resistivity reverse osmosis (RO) water were flushed through the filter with 103 kPa air, and the flushate containing removed particulates was collected in a volumetric flask. Numerous trials with known amounts of material loaded onto filters determined that using two sequential slugs of 125 mL of water, at 103-kPa air pressure, provided the highest systematic yield of filtered material (95 ± 5%). One of the four field samples was used to determine total suspended solids (TSS), and if that concentration was too small for particulate characterization, a composite of the other three was used to characterize the inorganic solids captured on the filters.

#### Dissolved metal/target analyte sampling

**Fig. 4:**
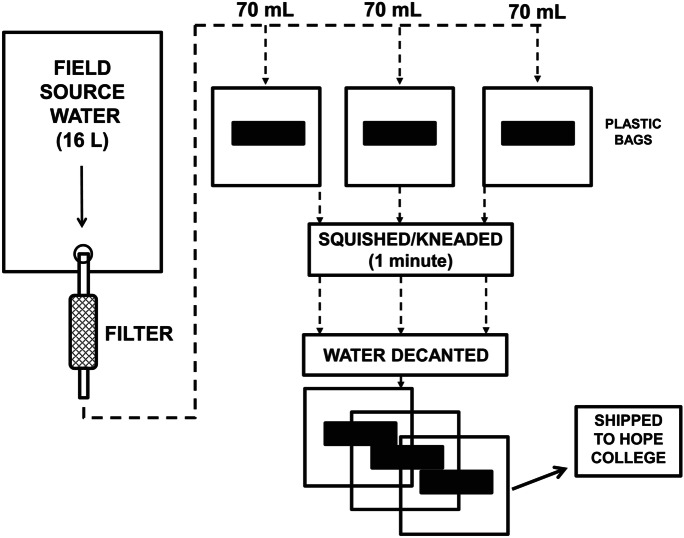
Schematic illustrating the field sample collection process utilizing polyurethane foam blocks (black rectangles) for the capture of dissolved analytes

Drinking water sources were sampled for dissolved analytes by using metal-capturing polyurethane foam blocks (Sawyer Products, Inc.) to sequester analytes from source water samples. A standardized field protocol was employed at all sampling sites (Fig. 4). Source water was first passed by gravity flow through the 0.1-μm hollow membrane filter to remove particulate matter, as described above. Three separate (70 mL) aliquots of filtered water were then placed in zip-sealed plastic bags along with a foam block. The foam was squished/kneaded with the water in the bag for 1 min, after which the water was decanted. The wet foam was then squeezed to remove residual water and shipped in the sealed bag back to Hope College for analysis.

#### Metal/target analyte retrieval

The returned field foams were processed through an acid-wash procedure to recover trapped analytes. Foam blocks were first dried at 100 °C for 24 h then weighed. Blocks were cut into two pieces and placed in a desiccator. Prior to rinsing, one half-block piece was reweighed and placed in an acid-washed 50-mL syringe equipped with a 0.45-μm syringe tip filter. Thirty (30) mL of 3% trace-metal grade nitric acid (TMG HNO_3_) (pH ~ 0.7) was added to the open syringe holding the foam, and the plunger was then inserted. After 5 min of foam-acid contact, the plunger was depressed, and the acid was filter-pressed through the foam block into an acid-washed, metal-free 50-mL conical tube. These rinsates were either analyzed immediately or refrigerated at 4 °C to preserve the integrity of the rinsates until analysis. Because the foam blocks employed for sequestering elements contained some of the target analytes as part of the foam formulation, rinsates from three separate batches, each consisting of 20 background control foams, were also analyzed. Foams were sent to the field in batches, and the average analyte concentrations from each batch of background control foams were correlated to field samples and used in the estimation of source water concentrations.

### Analysis

#### Back-flushed particulates (suspended load)

The suspended load present in source waters was estimated by spectrophotometry using a Microlab® FX522 system. The backflush sample was shaken vigorously, and a 7-mL aliquot was removed immediately for analysis. Attenuation (transmittance and absorbance) and scattering were measured at multiple wavelengths between 360 and 880 nm for 20 replicates. Relative error on attenuation measurements was < 1%. Samples were agitated vigorously between each replicate measurement.

**Fig. 5:**
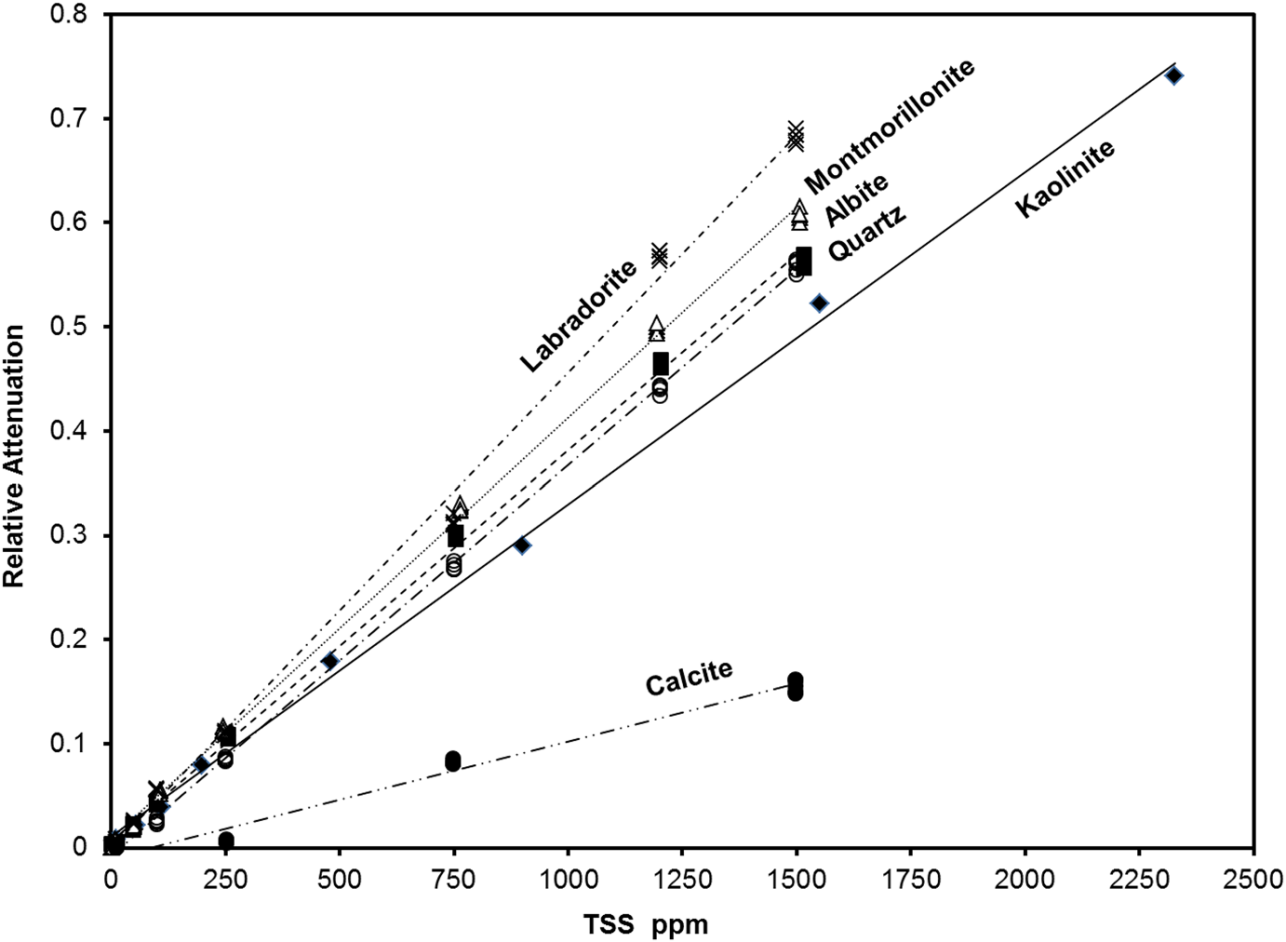
Standard curves of light attenuation versus TSS for model particulate suspensions. Correlation coefficients (R^2^) were > .99 for all curves except for calcite, which was .98

TSS concentration was estimated by comparison to standard curves of known suspended load (Fig. 5). Standard attenuation equations were developed for individual common rock-forming minerals which were considered representative of major types of geologic terrains, based on the assumption that the suspended load in any location is systematically reflective of the eroding substrate (Blake & Peters, 2015; Garzanti et al., 2011; Meybeck et al., 2003; Nasrabadi et al., 2018). All source sites were categorized as one of the following: plutonic, volcanic, metamorphic, or sedimentary. The representative minerals used were albite + labradorite (plutonic), labradorite + montmorillonite (volcanic), albite + montmorillonite (metamorphic), and quartz + kaolinite (sedimentary). Many of these minerals were identified in the particulate material recovered upon back-flushing. To be noted, “concentration” is a commonly used unit in this type of study, though samples are not solutions, sensu stricto, but rather suspensions. Mineralogy was determined and/or estimated by powder X-ray diffraction (PXRD) techniques (Rigaku® MiniFlex +) and SEM–EDS (Hitachi® TM-3000) analysis.

#### Metals/Analytes in rinsates (dissolved elements)

Rinsates were analyzed by ICP-OES techniques with a PerkinElmer® Avio 200 instrument. Analytes and detection wavelengths (nm) were As (193.69), Se (196.026), Zn (206.200), Pb (220.353), Cd (228.802), Ni (231.604), Fe (238.204), Mn (257.610), Cr (267.716), Mg (279.077), Cu (327.393), Ce (413.764), Sb (206.836), Ba (233.527), Se (203.985), and Cu (324.752).

**Fig. 6:**
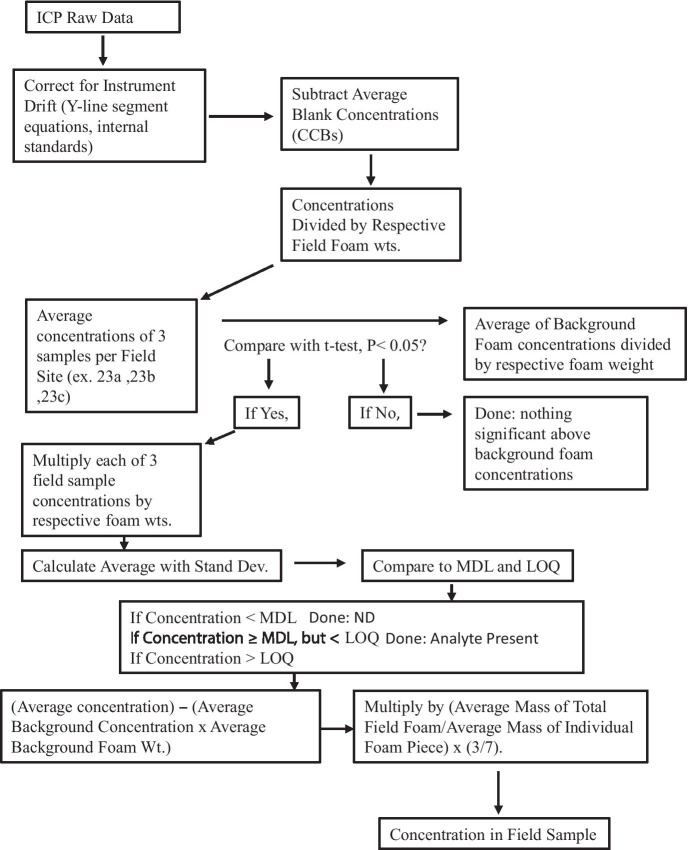
Processing flow chart scheme for estimating the field concentration of dissolved analytes in untreated drinking water sources based on the analysis of foam rinsates. MDL = method detection limit and LOQ = limit of quantification

Quality assurance (QA) and quality control (QC) checks were consistent with a modified protocol (EPA Method 200.7), as summarized in Sarojam (2010). Raw data were processed through a statistical comparison routine and reverse protocol algorithm to estimate target analyte concentrations in field drinking water sources (Fig. 6). Method detection limits (MDLs) and limits of quantification (LOQs) for analytes (μg/L, ppb) in this study were (MDL/LOQ): Ba (1.3/13), Cr (0.4/4), Mn (0.6/6), Fe (8.6/86), Ni (1.2/12), Zn (3.6/36), Cd (0.8/8), Se (26.1/261), As (7/70), Sb (3.7/37), Pb (1.5/15), Cu (1.1/11), Ce (4.5/45), and Mg (2.6/26).

Results reported from the analyte retrieval and analysis procedures were initially predicated on the assumptions: (1) All analytes present in the 70-mL field sample were transferred/bound to the foam in the plastic bag and (2) all analytes bound to the foam were rinsed off by flushing with 3% trace-metal grade nitric acid. If < 100% of the dissolved analytes were bound to the foam, and/or < 100% of the analytes were rinsed off the foam in the lab, then the final measured quantity was a minimum estimate of dissolved analytes in the drinking water source.

In order to test these assumptions, single-element foam retention/recovery testing was performed.

with 6 target analytes: Ba, Pb, As, Cd, Cr, and Cu. Aqueous solutions of 10, 20, 100, 200, and 1000 ppb of single-element analytes were mixed with foams, rinsed with acid and analyzed to mimic field and laboratory procedures. Results were compared to 0 ppb controls. Three different solutions from each experiment were analyzed. These were the input solution, the decanted supernatant after mixing, and the rinsate after filter pressing the foam with nitric acid.

Experiments revealed that an adjustment factor (AF) needed to be applied to measured foam rinsate concentrations in order to match input water concentrations. The AF was dependent on analyte and concentration ranges, with the smallest adjustments for all analytes (AF closest to 1.0) in the 10–100 ppb water concentration range, and the largest in the 100–1000 ppb water concentration range (Table 1). These results indicate that except for Ba, the assumptions of 100% analyte binding to the foam from source water, and 100% removal of analytes from the foam into the acid rinsate were not valid. Therefore, the target analyte concentrations determined in this study are minimum concentration levels for the source waters sampled in the field.

**Table 1:** Adjustment factors (AF) for foam rinsates based on laboratory retention tests

	Water concentration range (μg/L)					
Analyte	10–20	10–100	10–200	20–1000	100–1000	200–1000
As			6.5 (± 3.3)			9.7 (± 1.1)
Ba		0.7 (± 0.2)				2.3 (± 0.1)
Cd				3.2 (± 0.6)		
Cr	4.2 (± 2.7)				12.9 (± 2.7)	
Cu			1.8 (± 2.0)			3.3 (± 0.1)

As another check on foam binding, retention, and AF, field tests on local lake water (Holland, MI, USA) were performed in which direct analyses of water were compared to analyses of rinsates from lake samples subjected to the same field collection and rinsing protocols used in the global survey. An AF from foam retention tests could only be applied to the lake water tests for Cu, because this was the only analyte detected above the LOQ. Applying the AF to the foam rinsates resulted in a predicted Cu concentration of 45 μg/L (± 22 μg/L) compared to 11 ug/L (± 13 ug/L) measured in the water samples directly. These values are very near the LOQ for Cu of 11 μg/L. The lack of distinction between these concentrations (within 1 standard deviation) supports the AF approach and the conclusions that concentrations reported in this study should be considered minimum levels for the sources sampled.

There are some cautionary considerations regarding the laboratory foam retention tests and AF determinations. First, the tests were for single-analyte solutions, the behavior of which may not have reflected the processes active when multiple elements were present in solution with subsequent competition for adsorption sites on the foam. Also, the aqueous matrix of the test solutions (RO water) was not representative of the complex multi-constituent matrix of natural field waters.

## Results and discussion

### Particulates–suspended solids

Estimated particulate concentrations in the drinking water sources, as represented by TSS, are given in Table 2, along with qualitative characterization of the particulate constituents. TSS ranges from 0 to 92 mg/L (ppm), with 86% of the sources containing < 20 mg/L of suspended particulates. The percentage of particulates consisting of clays was estimated from X-ray diffractograms, and approximated to the nearest 5% by volume. Phases identified on PXRD were mostly quartz, feldspar, and clay, with a small number of samples containing metal oxides or sulfide phases. Results from SEM–EDS analysis are also shown in Table 2 as elements detected in a scan of the sample which are not directly attributable to clay, feldspar, or quartz phases, and are not other common rock forming elements (Al, Si, Na, K, Ca, Mg). Even though Fe is a common rock-forming element, it is separated out because of its potential affinity and association with As (Aredes et al., 2012; Catalano et al., 2011).

**Table 2:** Summary results

Site information				Site results												
Kit #	Country	Source type	Geologic terrain^+^	As	Ba	Cd	Cr	Cu	Ni	Pb	Sb	Se	Any analyte detectable, quantifiable or above WHO guideline?	TSS mg/L (± 5%)	Estimated % Clay^ƚ^	Particulate phase or composition detected other than clay, feldspar, quartz and RFE*
204	Brazil	River	S					P	P				Y	92	NM	Fe, Ti
205	Brazil	River	S	P				P	P				Y	73	25	Fe, Ti, Cu, S
206	Brazil	River	S						P				Y	91	NM	ND
189	Colombia	Tank	S										N	0.3	ND	ND
191	Colombia	River	S	P_rt_				P				P	Y	ND	ND	ND
141	Costa Rica	River	V			P							Y	1	25	Fe, Ti
142	Costa Rica	Tap	V	P_rt_									Y	0.3	25	Fe, Ti
143	Costa Rica	Tank	V	P_rt_									Y	0.4	50	Fe, Ti, Zn, S, Cl
180	Dominican Republic	Catchment	S	P_rt_									Y	58	25	Fe, Ti, Cl
181	Dominican Republic	River	S	P_rt_									Y	31	25	Fe, Ti
182	Dominican Republic	River	S										N	51	25	Fe, Ti
150	Ecuador	Rain Catchment	S	P_rt_									Y	0.6	ND	ND
151	Ecuador	River	S	P_rt_									Y	4	25	Fe, Cl, Ti
152	Ecuador	Unknown				P							Y	3	NM	ND
25	Fiji	Catchment	V										N	2	30	Fe, Ni, Cr, Cl, Ba, Ce
201	Guatemala	Tap	V	P	P			P	P				Y	ND	ND	ND
202	Guatemala	Well	V	P	P				P				Y	8	25	Fe, Ti, Cl
203	Guatemala	Tap	V	P	P			P	P				Y	ND	25	Fe, Cl, S
156	Haiti	Well Catchment	S										N	0.8	20	Fe, Ti, Cl, Zn
157	Haiti	Spring	S										N	4	25	Fe, Ti, Cl
158	Haiti	Well	S										N	2	20	Fe, Cl
177	Honduras	Tap	V			P							Y	2	25	Fe, Ti
178	Honduras	Tank	V										N	0.5	ND	ND
179	Honduras	Tank	V										N	2	25	Fe, Cr, Ti
11	India	Tap	S	P_rt_									Y	1	30	S, Mn, Fe
12	India	Well	S										N	1	25	Fe, Ce, Cl
13	India	Well	S										N	0.8	30	Fe, Ce
14	Indonesia	River	S										N	2	15	Fe, Cl
15	Indonesia	Well	S										N	0.7	25	Fe, Ti, S, Cl
16	Indonesia	Tap	S										N	0.6	0	Ce, S, Cl
174	Israel	Tank	S	P_rt_									Y	0.2	ND	ND
175	Israel	River	S	P_rt_									Y	ND	ND	ND
176	Israel	River	S	P_rt_									Y	0.1	ND	ND
147	Kenya	River	M										N	55	30	Fe, Ti
148	Kenya	Well		P_rt_									Y	0.3	ND	ND
149	Kenya	River		P_rt_									Y	2	20	Fe, Ti, Cl
168	Kenya	Well	V	P_rt_									Y	0.3	ND	ND
169	Kenya	Well	V	P_rt_									Y	0.8	40	Fe
170	Kenya	Catchment	V										N	74	25	Fe, Ti, Mn
8	Liberia	River	M	P_rt_									Y	1	ND	Fe, S, Ti, Cl
9	Liberia	Well	M										N	29	40	Fe, Ti, Cl
10	Liberia	Catchment	M	P									Y	29	40	Fe, Ti
5	Mexico	Tap	S	P_rt_									Y	0.1	ND	ND
6	Mexico	Tap		P_rt_									Y	0.4	ND	ND
7	Mexico	Tap	S	P									Y	3	25	Fe, Cr, Ni, Mn
153	Mexico	Well	Pl	P_rt_									Y	0.7	ND	ND
154	Mexico	Tap	Pl	P_rt_									Y	0.8	ND	ND
155	Mexico	Tap	Pl	P									Y	0.2	ND	ND
190	Mexico	Well	V	P_rt_									Y	0.4	25	Fe, Ti, Cl
192	Mexico	Tap	V	P_rt_									Y	0.1	ND	ND
196	Mexico	Lake	V	P_rt_									Y	5	25	Fe, Ti
162	Nicaragua	River	V										N	21	25	Fe, Ti, Cu
163	Nicaragua	Well	V	P_rt_									Y	19	25	Fe, Ti, Cl
210	Papua New Guinea	River	S	P_rt_	P		P	P_rt_					Y	4	25	Fe, Ti, Mn
211	Papua New Guinea	Catchment	S	P					P				Y	0.5	35	Fe, Cu, Zn
212	Papua New Guinea	River		P_rt_									Y	3	25	Fe, Ti
138	Peru	River	S									P	Y	8	30	Fe, Ti
193	Peru	River	S	P								P	Y	ND	25	Fe, Ti
194	Peru	River	S										N	ND	25	Fe, Ti
195	Peru	River	S	P_rt_ P_rt_									Y	ND	ND	ND
159	Senegal	Well	S	P_rt_									Y	0.8	25	Fe, Cl
160	Senegal	Well	S										N	2	0	Fe, S, Cl
161	Senegal	Well	S										N	2	5	Cl, S
186	Sierra Leone	Wetland	S	P									Y	0.6	35	Fe, Ni, Cl, S, Zn
187	Sierra Leone	Wetland	S	P									Y	0.1	40	Fe
188	Sierra Leone	Well	S										N	0.2	25	Fe, Cl
3	Solomon Islands	River	S	P_rt_				P_rt_					Y	2	25	Fe, S, Cl, Ti
4	Solomon Islands	Well	S										N	8	20	Cu, Ti, S, Fe, Mn
165	South Africa	Catchment	S	P_rt_									Y	0.3	ND	ND
166	South Africa	River	S	P_rt_									Y	3	25	Fe, S, Cl
167	South Africa	Unknown	S	P_rt_									Y	0.2	ND	ND

+Bedrock (CGMW, 1971; Choubert et al., 1988; De Wit et al., 1988; Meuhlberger, 1992; Kirkham et al., 1995)

*S* sedimentary, *V* volcanic, *M* metamorphic, *Pl* plutonic

*Relative %, PXRD analysis (*ND* not determinable, *NM* not measured)

*Rock forming elements (Al, Si, Na, K, Ca, Mg); *P* analyte present, *P*_*rt*_ analyte quantifiable

**Fig. 7:**
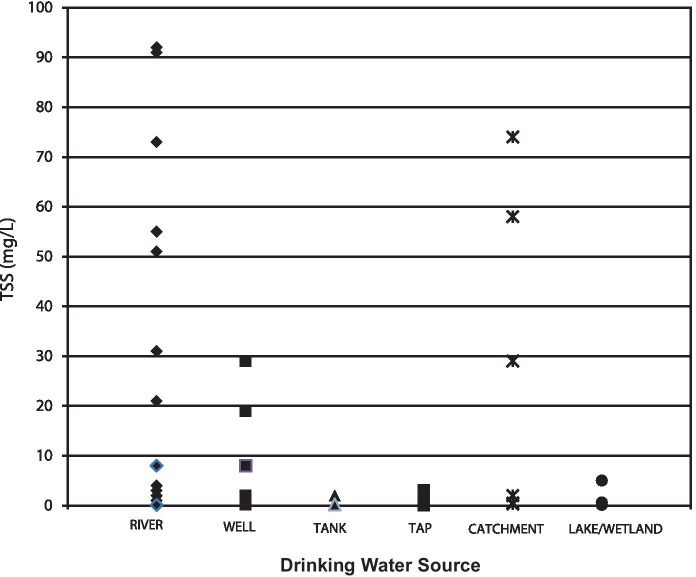
TSS results for different types of untreated drinking water sources sampled in this study

Figure 7 is a comparison of the estimated TSS results with the various drinking water sources sampled in the study. River sources had the largest range of TSS, with the highest values approaching 100 mg/L. These levels were similar to other rivers studied and would be considered relatively low TSS (Meybeck et al., 2003), with higher concentrations commensurate with faster-flowing streams with higher bed shear stresses (Alkhatib & Berna, 2008), terranes dominated by unconsolidated geologic material (Cagnin et al., 2017), substrate disruptive anthropogenic activity (Nasrabadi et al., 2018), or a combination of these factors. Catchments in this study consisted of sequestered water within earthen basins or impoundments, a reason the ranges of TSS were similar to river concentrations. Suspended solids of groundwater wells was expected to be low because of the relatively low linear velocity of flow through an aquifer; however, values ranging over 4 orders of magnitude 10^0^–10^3^ mg/L have been reported (Degueldre et al., 2000; McDowell-Boyer et al., 1986; Peterson et al., 2019), indicating 1–30 mg/L measured in this study was within the typical range. High levels of groundwater TSS reported could be the result of non-ideal well design or installation, and/or reflect aquifer matrices containing a significant proportion of particles which are suspendible only upon pumping or bailing (Peterson et al., 2019).

Particles were mostly composed of clays (kaolinite, illite, and montmorillonite), feldspars, and quartz. This mineralogy, along with the relative proportion of clays (Table 1), were both consistent with the suspended load mineralogy of the near surface water of the Ganga River, considered one of the largest representative models of suspended load in the world (Garzanti et al., 2011). The elemental compositions of particulate phases which could not be identified as specific minerals are also listed in Table 2. The presence of Fe, Ti, Cu, Zn, Ni, Cr, Ba, and Mn reflects the progressive enrichment of elements contained in, or adsorbed to, slow-settling clays or oxyhydroxides (Garzanti et al., 2011). These elements are commonly reported constituents in drinking water sources in the developing countries (Chowdhury et al., 2016). Sulfur and chloride are indicative of ore-phases of the metal or metalloid elements, and the detection of the light rare earth element (LREE) Ce suggests the presence of allanite, monazite, or other LREE-bearing minerals.

### Dissolved metals and other analytes

**Fig. 8:**
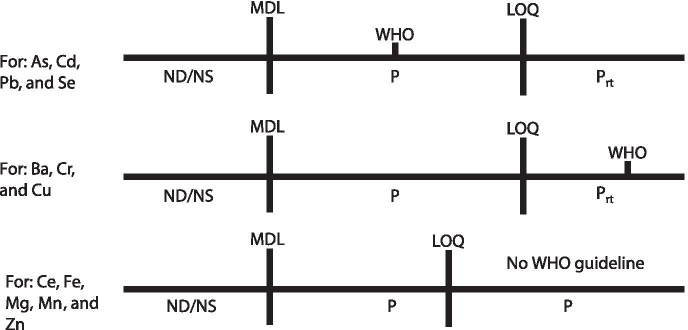
Categorization scheme for various target analyte concentrations determined in samples. MDL = method detection limit and LOQ = limit of quantification. *ND/NS* = non-determinable/not significant; *P* = present, but not quantifiable; *P*_*rt*_ = quantifiable; WHO = World Health Organization guideline

Results were compiled into 3 categories for each analyte for every site (Fig. 8). These categories were non-determinable/not significant (ND/NS), present (P), and present with recommendation for re-testing (P_rt_). ND indicates that the analyte concentration was below the method detection limit (MDL) for the protocol and instrument used in the study. NS indicates the analyte concentration was statistically indistinguishable from the composition of the background control foam rinsates. P indicates that concentration was above the MDL but below the limit of quantification (LOQ), or above the LOQ with no WHO guideline. P_rt_ indicates that the analyte concentration was quantifiable (> LOQ) and either below or above the WHO guidelines (As = 10 μg/L, Ba = 1300 μg/L, Cd = 3 μg/L, Cr = 50 μg/L, Cu = 2000 μg/L, Ni = 70 μg/L, Pb = 10 μg/L, Sb = 20 μg/L, Se = 40 μg/L) for the contaminant (WHO, 2017). It would be recommended that these water sources be retested directly for the field concentration of the dissolved analytes.

Table 2 includes a summary of the results, showing that 68% of the sites sampled contain analytes which are detectable or quantifiable (P and P_rt_), and 67% would be recommended for retesting (P_rt_). Only the analytes with a WHO guideline are listed. Fourteen percent (14%) of the sites have more than one detectable analyte present. Cu and As are the only target analytes which occur above the WHO guideline, with As exceeding the guideline in 45% of the sources sampled. Other metals (with no WHO guideline) were detected in the sources, with maximum concentrations of Ce reaching approximately 3500 μg/L, Fe 260 μg/L, Mg 13,000 μg/L, Mn 1600 μg/L, and Zn 94,000 μg/L. Overall, the most commonly occurring dissolved metal found in the drinking water sources was Mn.

**Fig. 9:**
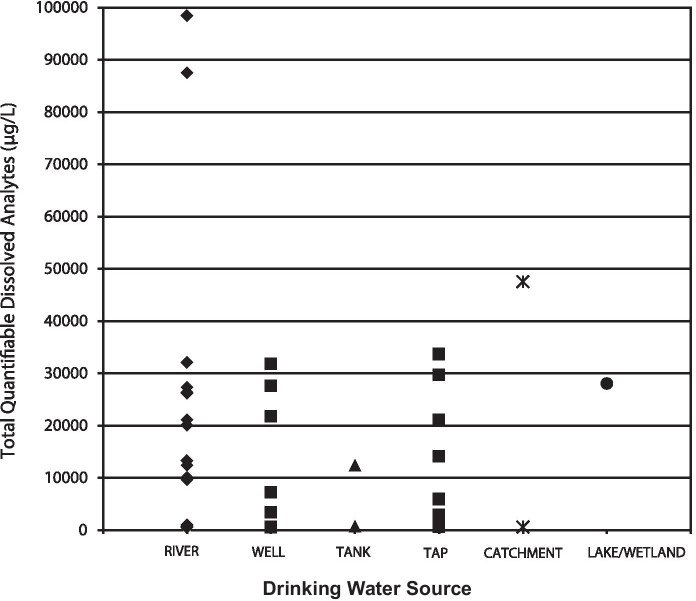
Total quantifiable dissolved analytes estimated in drinking water sources. Totals represent the sum of all dissolved analytes detected at concentrations above the respective LOQs

Figure 9 is an illustration of the total quantifiable dissolved analytes (TQDA) measured for each of the drinking water source types. TQDA is the sum of the analyte concentrations which occurred above the LOQ in the sample. These included the analytes for which a WHO guideline has been established (Table 2) plus metals such as Fe, Zn, Mn, and Mg, for which no guideline exists. Except for two very high values near 90,000–100,000 μg/L, the majority of the samples peaked just above 30,000 μg/L, or 30 ppm.

**Fig. 10:**
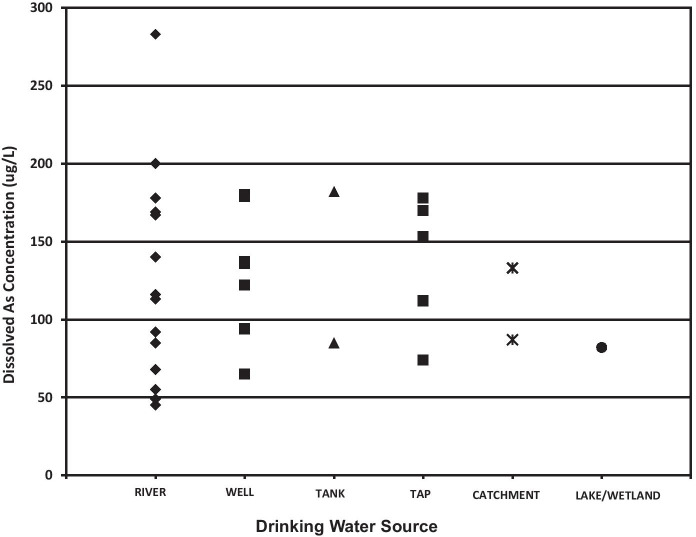
Dissolved As concentrations estimated for the drinking water sources sampled

Dissolved As concentration compared to source type is shown in Fig. 10. Except for one high value of approximately 285 μg/L, maximum dissolved As concentrations of about 200 μg/L, and concentration ranges of 45–200 μg/L (river), 65–179 μg/L (well), and 112–178 μg/L (tap) were all similar. All of these values are above the WHO guideline of 10 μg/L and are comparable to the groundwater As concentrations (maximum 134 μg/L) measured in hand tube-wells from some regions of Bangladesh (Chakraborti et al., 2015). Other studies have reported similar orders of magnitude for As in drinking water from Croatia (Ćavar et al., 2005), Iran (Mosaferi et al., 2008), Pakistan (Baig et al., 2010), and many Latin American countries (Bundschuh et al., 2012).

**Fig. 11:**
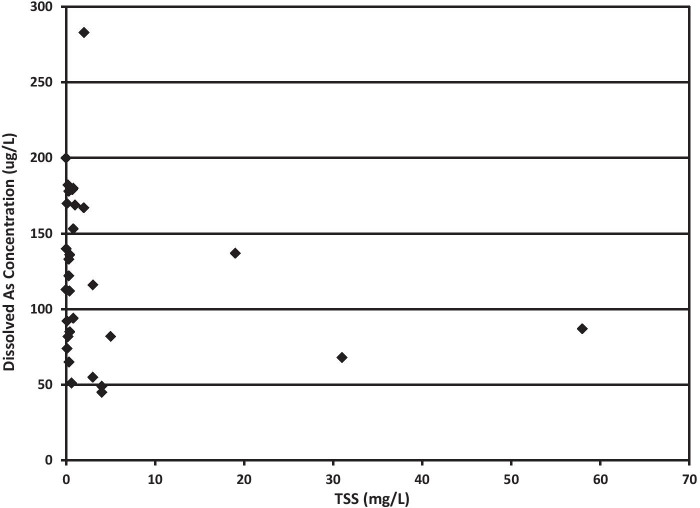
Dissolved As concentrations versus TSS in water sources with quantifiable As levels

No observable correlation was found between TSS and As concentrations (Fig. 11) in this study. This should be expected because the sources and source type vary. Nor does a correlation exist between As concentration and TSS when samples from the same source type were compared. In other studies, relationships have been observed in locations where As-bearing bedrock and As-bearing suspended particulates occur (Blake & Peters, 2015; Cagnin et al., 2017; Nasrabaldi et al., 2018); however, the results of several studies (Baig et al., 2010; Cagnin et al., 2017; Costas et al., 2011; Grosbois et al., 2011) indicate the mineralogical composition of the suspended particulates has the largest control on dissolved As concentration, specifically controlled by redox chemistry of Fe and Mn oxides and oxyhydroxides. In the current study, Mn concentrations were very consistent among all samples with quantifiable As present, averaging 476 ppb ± 33 Mn. It was not possible to evaluate a relationship with Fe concentrations because in the majority of samples analyzed Fe concentrations were not statistically distinguishable from the Fe levels measured in background foam rinsates.

To the authors’ knowledge, this study was the first attempt to perform a global reconnaissance survey of untreated drinking water sources using small-volume, point-of-use drinking water filters and foams, for the primary purpose of characterizing particulates and dissolved contaminants in water. Previous studies (Mull & Hill, 2012; Smith & Hill, 2009) have utilized dialysis filters successfully for the collection of microbes from water, and some investigations (Grytdal et al., 2018; Knappett et al., 2011) used this approach to survey a defined population who access multiple sources of drinking water, within a relatively small geographic region. The global scale and systematic protocol of the current study, from sampling through analysis, made this a potentially robust and cost-effective approach.

The field use of polyurethane foams to sample and sequester potential dissolved metal and other contaminants was useful as a reconnaissance tool by providing minimum concentration levels. Only minimum concentration levels could be estimated because of the likelihood of incomplete capture efficiency and retrieval in sampling and processing, as indicated by the results of laboratory single-element retention tests which indicate less than 100% analyte recovery from acid rinsing of spiked foams. The composition and compositional variability of the foam blocks required average background subtraction of concentrations of dissolved species which were both target analytes and constituents of the polyurethane foams. This means that copper, iron, and some other base metals may have been present at relatively high concentrations in the water sources, but were statistically indistinguishable from the concentrations in background (control) foam rinsates.

The classification of results for analyte concentrations, as presented in this paper, should be useful for disseminating reconnaissance data to stakeholders in local, regional, or national water quality decision-making venues. An initial indication that a water source has a metal or other contaminant present, but not above WHO guidelines, as opposed to present and above WHO guidelines will help triage sites for further investigation and remedial action. This qualitative indicator approach may be more useful than a quantitative value because any single sampling event represents only a snapshot in time—a numerical value may give a false impression of accuracy.

## Conclusions

This study demonstrated that hollow-membrane point-of-use water filters and metal-capturing polyurethane foams can be used for a reconnaissance characterization of TSS and dissolved analyte concentrations in untreated drinking water. The project design of sending sampling kits to different countries to be used by trained NGO personnel, with non-technical backgrounds, for water sampling is a potentially powerful approach to accomplish a global survey. Results obtained for TSS and analyte concentrations were comparable with published results (Degueldre et al., 2000; Meybeck et al., 2003; Cavar et al., 2005; Alkhatib & Berna, 2008; Mosaferi et al., 2008; Baig et al.,  2010; Bundschuh et al., 2012; Chakraborti et al.,  2015; Chowdhury et al.,  2016; Cagnin et al.,  2017; Nasrabadi et al.,  2018; Peterson et al.,  2019) reported from other locations where water was tested directly. Less than complete removal of target analytes from source waters in combination with incomplete recovery of analytes from off the foams during acid rinsing limited the reporting of analyte concentrations to minimum concentrations in the drinking water sources. A methodology of reporting analyte concentrations as present but below WHO guidelines, or quantifiably present and above WHO guidelines, with the recommendation to retest, may be a useful triage tool for decision-makers responsible for water-quality investigations or remediation, resulting in a more strategic allocation of resources.

## Data Availability

The data used and analyzed which support the findings of this study are available from the corresponding author upon reasonable request.
